# Understanding Responsible Development in AI-Based Clinical Prediction Models for Mortality: Protocol for a Scoping Review

**DOI:** 10.2196/80325

**Published:** 2026-03-05

**Authors:** Riley Martens, Jessalyn K Holodinsky, Jessica Simon, Zack Marshall

**Affiliations:** 1 Department of Community Health Sciences, Cumming School of Medicine, University of Calgary, 3280 Hospital Dr NW, Calgary, AB, T2N 5A1, Canada, 1 (403) 220 6940; 2 Department of Emergency Medicine, Cumming School of Medicine, University of Calgary, Calgary, AB, Canada; 3 Department of Clinical Neuroscience, Cumming School of Medicine, University of Calgary, Calgary, AB, Canada; 4 O'Brien Institute for Public Health, Cumming School of Medicine, University of Calgary, Calgary, AB, Canada; 5 Department of Oncology, Cumming School of Medicine, University of Calgary, Calgary, AB, Canada; 6 Department of Medicine, Cumming School of Medicine, University of Calgary, Calgary, AB, Canada; 7 School of Social Work, McGill University, Montréal, QC, Canada

**Keywords:** artificial intelligence, AI, responsible development, mortality prediction, responsible research and innovation, RRI, machine ethics, patient engagement, sociotechnical, knowledge synthesis

## Abstract

**Background:**

Prognostic inequity has been identified as a barrier to accessing end-of-life care for underrepresented groups. Artificial intelligence–based clinical prediction models (AIPMs) for prognostication of mortality have the potential to offer rapid, accessible, and accurate predictions that could streamline care. However, they may also exacerbate preexisting inequities in the health care system rather than address accessibility and quality. This can be caused by erroneous outputs from biased training data, outcomes from out-of-scope operationalization, and inexplicability due to opacity.

**Objective:**

The goal of this study is to synthesize peer-reviewed literature on the creation and application of AIPMs to prognosticate mortality in acute care settings for adult patients, offering new insights into responsible and ethical model development.

**Methods:**

A transdisciplinary, structured search strategy was developed in consultation with librarians from both health sciences and engineering sciences. The academic databases queried were Medline, Embase, IEEE Xplore, ACM Digital Library, Compendex, and Scopus. The search was conducted in spring 2025, and the results were uploaded to Covidence. A team of reviewers will screen in 2 rounds: titles and abstracts, then full texts. Eligibility will be determined by publication in academic journals or as full-length conference proceedings, language, model output, and AI use. Data will be charted using adapted charting tools and then analyzed by descriptive, summary, and qualitative synthesis.

**Results:**

The search was completed on March 25, 2025, with screening starting in May 2025. Results are anticipated for January 2026.

**Conclusions:**

This review will provide a comprehensive summary of AIPMs that predict mortality, highlighting the specific elements included in their development. Informed by the responsible research and innovation (RRI) framework, we will consider interest-holder engagement, interdisciplinary collaboration, and computational and clinical ethics will in the context of the four RRI dimensions: anticipation, reflexivity, inclusion, and responsiveness.

## Introduction

### Background

Artificial intelligence–based clinical prediction models (AIPMs) have been developed in a range of medical disciplines to predict the outcomes of different diseases [1]. For example, cardiologists have developed AIPMs to predict mortality in heart failure, and neurologists have trained an AIPM to examine stroke outcomes [2,3]. Another tool, NYUTron, a large language model, showed considerable improvement compared to non-AI CPMs in determining in-hospital mortality for new admissions [4]. AIPMs have demonstrated the ability to predict mortality for patients across various conditions [2-4]. The availability of accurate and timely prognostication could provide tangible benefits to patients, clinicians, and health systems.

AIPMs also have the capacity to exacerbate preexisting inequities in the health care system instead of improving accessibility and equality [5]. This can be caused by erroneous outputs from biased training data, unintentional outcomes from out-of-scope operationalization, and inexplicability due to opacity [5-7]. Training data contain biases from disproportionate representation of some sociodemographic groups [5]. Using AIPMs beyond their intended purposes poses risks because of knowledge limits hardcoded into these tools [7]. Further, low AI literacy can influence patient engagement unfairly and may discourage interest holders from future involvement [6]. Responsible development relies on guiding principles for the design of technology that prioritizes harm reduction and prevention [8]. To safeguard against the exacerbation of inequities, responsible development is an encouraged approach to AIPM creation.

The responsible research and innovation framework (RRI) outlines 4 important components of accountable research [9]: anticipation, reflexivity, inclusion, and responsiveness. Each of these items tied to recommended action points [9]. Anticipation warrants foresight and future-oriented assessment, which can be impacted by limited scopes and siloed disciplines [9,10]. A proposed way to overcome these barriers would be to incorporate a transdisciplinary research approach [10]. Reflexivity encourages critical self-appraisal and evaluation of one’s methods and work [9]. Standards and frameworks have been applied to encourage reflexivity, including guidelines regarding development [9]. Inclusion leads to engagement with communities and interest holders who have not always been involved in research [9,11]. This highlights the strategy of public engagement and interest holder consultation [6,11]. Responsiveness, in contrast to the other three aspects, requires extended action to answer questions, beyond information seeking [9]. Responsiveness includes reacting and changing the course of work based on feedback and implications [9]. In relation to AIPM development, impact assessment would be an apt strategy to address responsiveness [1].

This review benefits from the structure of responsible development and respective action items to explore AIPMs. Previous reviews have been conducted of AIPMs, typically highlighting technical performance or a particular pathology [1-3,12]. This review is differentiated by two elements: the responsible development lens and the transdisciplinary approach. This review will examine any AIPMs that use mortality as the primary outcome regardless of machine learning technique or specific pathology. Components of AIPM development will be explored, including interest holder engagement, interdisciplinary collaboration, and ethical frameworks. AIPMs developed for acute care contexts will be the focus, including in emergency rooms, critical care, or other in-patient medical units, as that is representative of the majority of these tools.

The objective is to synthesize peer-reviewed literature on the creation and application of AIPMs to predict mortality in acute care settings with adult patients, offering new insights into responsible and ethical model development.

### Review Questions

This review will answer four questions regarding AIPMs predicting mortality. First, how are AI, outputs, and performance reported when describing the development of AIPMs? Second, in the acute care settings the AIPMs are developed and deployed in, what is their intended use, and how are they evaluated? Third, how have responsible research and innovation considerations been integrated into the development of these AIPMs? Do researchers report on ethics, interest-holder engagement, interdisciplinary collaboration, or other elements of responsible development? Fourth, what are the patterns, advances, gaps, evidence, and recommendations (ie, the elements of the PAGER framework) in the literature regarding the responsible development of AIPMs in predicting mortality [13]?

## Methods

### Overview

This scoping review will follow the extended Arksey and O’Malley [14] framework described by Levac et al [15] through six stages, from identifying the research question to consultation with interest holders. The protocol is formatted according to the JBI template for clear reporting [16]. The protocol will be submitted for publication to support the reproducibility and transparency of the review, pending anticipatory results [17]. Study eligibility will be determined using preestablished criteria. The results will be reported using the PAGER framework to highlight patterns, advances, gaps, evidence, and recommendations [13]. Additionally, this review will follow the PRISMA-ScR (Preferred Reporting Items for Systematic reviews and Meta-Analyses extension for Scoping Reviews) checklist to ensure all necessary elements are included [18].

### Inclusion Criteria

Formatting the objective into the population, concept, and context elements, as recommended by the JBI guidelines [19], the review objectives are stated in Table 1.

**Table 1.** Research objective in the population, concept and context format.

Element	Description
Population	Artificial intelligence–based clinical prediction models that predict mortality
Concept	Examining definitions, concepts, and elements of responsible development
Context	Artificial intelligence–based clinical prediction models used in acute care settings with adult patients

#### Population

Clinical prediction models that use machine learning or other methods that are considered to be AI and that provide mortality as the primary outcome will be the population for this review. This excludes any models that only use statistical techniques or provide outcomes other than mortality. Novel machine learning applications will be the focus, whereas preexisting clinical tools or external validation methods will not be considered. AI and machine learning in this review will considered to be supervised, nonparametric predictive modeling techniques with flexible, nonlinear relationships between predictors and a mortality outcome. These approaches include decision tree–based and ensemble algorithms, which differ from traditional regression models that impose linearity or distributional assumptions and from unsupervised clustering methods used for risk stratification.

#### Concept

Responsible development is the concept being examined, as defined by preset criteria, including interest-holder engagement, interdisciplinary collaboration, and computational and clinical ethics [20,21].

#### Context

AIPMs used in acute clinical contexts for adult inpatients in emergency rooms, in-hospital units, and critical care will be eligible. Any studies conducted in community settings, outpatient care, or pediatric populations will be excluded.

### Types of Sources

All study designs will be considered, including experimental, quasi-experimental, qualitative, and mixed methods research. The sources deemed relevant to this review will be peer-reviewed articles published in scientific journals or full-length conference papers. Eligibility will be determined based on the research objectives and outputs reported. No language or date filters will be applied. Systematic reviews and scoping reviews will not be included as full texts but will be identified during the title and abstract screening phase. This is to ensure the quality and robustness of this review by further investigation of reference lists from other relevant reviews.

Any work not published in a scientific journal or in an academic conference proceeding will be excluded, such as gray literature, white papers, or opinion papers. This extends to any editorials or position papers where no development of a machine learning application occurred.

### Search Strategy

A transdisciplinary, structured search strategy was developed in consultation with a health sciences librarian and an engineering sciences librarian. Precursory searches in EMBASE and Medline informed the identification of index terms. This was accomplished by using Yale’s MeSH Analyzer combined with adapted search filters from the database of the CDA-AMC (Canada’s Drug Agency–L’Agence des médicaments du Canada) and the University of Alberta’s field-specific search resources [22-25]. A sample search strategy for Medline is shown in Multimedia Appendix 1. Four concepts related to the responsible development of prognostic AIPMs shaped the search strategy based on previously published search filters [22-24]. The first search concept includes any clinical decision support terms to capture the type of AI tool being developed, including clinical prediction models. The second concept in the search strategy relates to artificial intelligence and uses terms related to neural networks and machine learning. The third concept in the search focuses on mortality, including terms such as “death” and “end-of-life.” The fourth concept reflects the acute setting in which the AIPMs are used and includes the terms “emergency department,” “critical care,” and “inpatient units.”

The academic databases selected for this review are Medline, Embase, IEEE Xplore, ACM Digital Library, Compendex, and Scopus. Medline and Embase were chosen to capture any AIPMs published in health care journals. IEEE Xplore, ACM, and Compendex were chosen to collect literature from the engineering and computer science fields. Scopus was selected as the interdisciplinary database.

### Selection of Studies and Sources of Evidence

After the consolidation and execution of the search strategy across databases, the results will be uploaded to Covidence [26]. Covidence’s deduplication tool will be used to remove identical studies. There is potential for a large return of studies given the search strategy. To address this, screening will be done in stages in addition to the use of screening software, Covidence, and a team of multiple screeners. A team of secondary reviewers will begin the first round of screening on the search results. Pilot screening will occur in rounds, with batches of 100 studies, to assess rater agreement, with additional pilot screening to increase agreement as needed to reach an interrater agreement of 75% between the secondary team and primary reviewer. Overall, interrater agreement will be tracked automatically through Covidence, with conflicts being resolved through discussion and consensus. Similar inclusion and exclusion criteria will be applied in the second round of screening, when reviewers progress to full-text review (Multimedia Appendix 2 provides the detailed inclusion and exclusion criteria). For full-text review, a proportionate agreement of 75% will also be agreed a priori with a pilot screening round of 25 studies. If the agreement rate does not hold after 25 studies, it will be repeated. The exclusion criteria, including gray literature and preprints, were chosen to define and synthesize peer-reviewed research. As the subject matter of AI and machine learning is rapidly evolving, the focus of the review was academic publications and the model development being reported. An updated search in the future will capture any preprints that are peer-reviewed and published.

The exclusion of full texts that are not in English should occur in the title and abstract round of screening, given the language criteria. If there are any non-English texts present in the subsequent screening round, they will be removed according to the eligibility criteria.

Any differences will be resolved through discussion and consensus. Upon completion of two rounds of screening, the studies relevant to the research question will progress to data charting. The review process will be illustrated in a PRISMA flow diagram when reported [18].

### Data Charting

Following full-text review, studies will be stratified based on the level of RRI involvement. If the studies report no elements, limited charting will occur, but if the studies report multiple RRI elements, then full data charting will be done. Information from included studies in the scoping review will be charted by two independent reviewers using a data collection tool developed by the team. The data charting form includes adapted criteria from the TRIPOD (Transparent Reporting of a multivariable prediction model for Individual Prognosis Or Diagnosis)+AI checklist, the EQUALSS (ethnicity and race, qualifications and education, underserved area, age, language and religion, sex, sexual orientation) framework, the artificial intelligence risk management framework, and other guiding principles [27-30]. Data will include specific details about study participants, machine learning techniques, clinical settings, and impact assessments relevant to responsible development.

RRI principles of anticipation, reflexivity, inclusion, and responsiveness have informed the formation of the review and thus structured the data charting and subsequent analysis. Anticipation will be mapped to the level of transdisciplinarity of eligible studies. This will be assessed through any report of multidisciplinary, interdisciplinary, or transdisciplinary collaboration in addition to the academic composition of the study team. Reflexivity will correspond to any standards or frameworks implemented in the work, including best practices, ethical frameworks, and other relevant standards. These will be collated under charting fields and further refined into research, ethical, and clinical practice standards. Further, inclusion correlates to interest-holder involvement, especially patient and family engagement. This will be assessed using the Patient-Oriented Research Level of Engagement Tool (PORLET) [31] to determine level and extent of engagement. This will be followed by responsiveness and data charting relevant to this principle and impact assessment, assessed as the impact a tool has on clinical care, including fairness in patient demographics (determined by EQUALSS) and any reported environmental impacts.

A draft charting form is provided in Multimedia Appendix 3. Pilot data charting by at least two reviewers is planned for the first five eligible studies. For the data charting phase, the calibration of complex topics will involve a charting template with preestablished criteria and tools including TRIPOD+AI, PORLET, and EQUALSS. Tandem charting will occur, where multiple screeners will chart information from eligible studies followed by verification by another screener. If there are differences in the elements charted, adjudication by a third screener, the supervisor of the project, will occur.

The data charting tool will be iteratively revised as necessary during the process of extracting data from each included evidence source. As part of the subsequent analysis, after charting there will be a tally of the elements present that fulfill the RRI principles, including TRIPOD+AI, PORLET, EQUALSS, and the presence of fairness metrics. This tally will be calculated based on the presence of RRI elements, scores from TRIPOD+AI compliance, and PORLET. Any modifications will be detailed in the review.

### Data Analysis and Presentation

The RRI principles have informed the elements for data charting, but PAGER provides the structure for the reporting of the charting. The patterns section will detail the most prevalent elements numerically across the four RRI principles. Advancements will showcase the development of the RRI principles chronologically based on date of publication. Gaps will be demonstrated by any anomalies that are present in patterns or advancements. Evidence for practice will be synthesized based on the charted elements that had the highest prevalence of RRI principles and prognostic accuracy. Additionally, any recommendations from authors of eligible studies will be collated. Recommendations for research will be synthesized based on observed gaps and charted data by the study team.

Adhering to the PAGER framework, a patterning chart will be used to organize and visualize advances and gaps in the evidence [13]. Additionally, this will be accompanied by descriptive tabulations and co-occurrence analysis [32]. This will be done to further identify the frequency of each charted element and the overlap between them [32]. Co-occurrence will follow a deductive approach using conceptual codes from the data charting forms. This process will further identify which concepts appear together or appear in isolation, as well as overarching themes.

A narrative summary will follow to highlight the prevalent aspects of the population, concept, and context, which includes machine leaning models, responsible development, and clinical settings [13,19]. An additional component will include an analysis of the chronology of the publications. Through these analyses, the considerations and implications of responsible development of AIPMs predicting mortality will be made clear.

## Results

The search for this study was conducted on March 25, 2025, and screening commenced in May 2025 with anticipated reporting by January 2026. The initial search returned 9475 results after deduplication, and the search will be updated in summer 2025 to ensure that newly published literature is assessed in this review.

## Discussion

### Anticipated Findings

AIPMs have the potential to improve prognostication, but without attention to responsible development, they could also amplify inequities [5]. Certain ethical criteria have been highlighted in the use of AI, including technological literacy, patient engagement, and explainability [6,33]. Reviews or other studies exploring the ethical dimensions of AIPMs typically do so in isolation [6,33]. The exploration of these aspects in combination is a novel facet of this review.

Palliative care is often seen as one of the areas least impacted by AI due to its relational nature and specific focus on the care continuum [34]. New ethical precedents such as surrogate decision-making and goals of care have been established with the introduction of novel technology in end-of-life care, as demonstrated by feeding tubes and ventilators that can prolong life beyond a time that the patient can consent or decline certain treatments [35]. AIPMs may significantly impact palliative care practices by reducing some of the uncertainty in prognostication. With growing interest in large language models and agentic AI, this is an opportune time to codify responsible development [34,36,37].

Palliative care must be patient-centered, as patients and families choose between different care pathways [35]. Information, education, and transparency empower patients to make decisions based on their values and priorities. Opacity and low digital literacy are barriers to patient-centered care in this context, as any AIPM providing prognostic information to a clinician can influence care [6,38]. Opacity means these models are not easily understood, nor are they explainable in their functioning, resulting in a clinician potentially needing more technical knowledge to critically evaluate their output [39]. Additionally, the digital literacy levels of the clinician, patient, and family can hinder decision-making when using or attempting to use information from these tools [6,33]. Clinicians may not be able to explain how the information is derived, and as a result patients and families may not be familiar or comfortable with the technology.

### Limitations and Strengths

As machine learning and AI are trending topics, the growth in literature is rapid [37]. Timeliness is one limitation to the relevance of this review’s findings, as new guidelines, frameworks, or outlines determining responsible development continue to be developed and introduced [29,40]. Though the inclusion of seven databases should yield strong interdisciplinary results, the noted absence of CINAHL does represent a limitation of this review. We considered excluding PSYCInfo and Web of Science due to their overlaps with other databases, notably Scopus and Medline. We excluded CINAHL to emphasize the technical and biomedical literature, which are more present in other databases. When we obtained nearly 10,000 results, we made the decision to finalize the databases. Limitations related to databases and the timeliness of the review, given the rapidly evolving subject matter, will be addressed in an updated search. Changes in technology could also undermine the salience of the results, as a potential shift from prediction models to large language models or agentic AI could take place. This makes up-to-date knowledge synthesis in this area particularly challenging. The emphasis on ethical elements during development will help inform future work in this area. This is why focusing on concepts in responsible development, including interest holder engagement, interdisciplinary work, and ethics, will make it more likely that this review will remain timely despite changing regulations, frameworks, or technology. In addition, this review will highlight critical concepts related to responsible development across medical specialties for AIPM in acute care. To avoid perpetuating prognostic inequities, exploring the extent of responsible development in AIPM could help identify and reduce concerns related to bias, inexplicability, and consent [9].

## Supplementary material

10.2196/80325 Multimedia Appendix 1 Search strategy.

10.2196/80325 Multimedia Appendix 2 Inclusion and exclusion criteria for screening.

10.2196/80325 Multimedia Appendix 3 Data charting form.

10.2196/80325 Multimedia Appendix 4 Decision tree for artificial intelligence model eligibility.

## Abbreviations

AI: artificial intelligence
AIPM: artificial intelligence–based clinical prediction model
CDA-AMC: Canada’s Drug Agency–L’Agence des médicaments du Canada
EQUALSS: ethnicity and race, qualifications and education, underserved area, age, language and religion, sex, sexual orientation
PAGER: patterns, advances, gaps, evidence, and recommendations
PORLET: Patient-Oriented Research Level of Engagement Tool
PRISMA-ScR: Preferred Reporting Items for Systematic reviews and Meta-Analyses extension for Scoping Reviews
RRI: responsible research and innovation
TRIPOD+AI: Transparent Reporting of a multivariable prediction model for Individual Prognosis Or Diagnosis + AI
