# Supplementary material for: Understanding Responsible Development in AI-Based Clinical Prediction Models for Mortality: Protocol for a Scoping Review
Source: JMIR Res Protoc. 2026 Mar 5;15:e80325. doi: 10.2196/80325 (PMC12978964; doi:10.2196/80325)
Supplement: Multimedia Appendix 1 [file resprot-v15-e80325-s001.docx]

### Appendix I: Search Strategy in Medline on March 25th, 2025

Concept 1:

exp Decision Support Systems, Clinical/ or exp Decision Support Techniques/ or exp Models, Statistical/ or exp Prognosis/ or ("clinical prediction model" or "clinical decision support" or "decision support system" or predicting).tw,kf.

Concept 2:

exp Artificial Intelligence/ or exp Machine Learning/ or exp Pattern Recognition, Automated/ or exp Neural Networks, Computer/ or exp Algorithms/ or (AI or "artificial intelligence" or AIVI or "classification algorithm*" or "computer heuristic*" or "convolutional network*" or "decision tree" or "deep learning" or "data science" or "feature detection" or "generative pre-trained transformer" or "generative pretrained transformer" or "language learning model*" or "large language model*" or "learning algorithm*" or "machine learning" or "machine intelligence" or (Markov adj3 model*) or ((multifactor* or multicriteria) adj3 ("decision analysis" or "decision making")) or "natural language process*" or "nearest neighbo*" or "neural network*" or "outlier detection" or "pattern recognition" or "probability tree" or "random forest" or "representation learning" or "support vector machine*" or "transfer learning").tw,kf.

Concept 3:

exp Death/ or exp Hospital Mortality/ or exp Mortality/ or (death or deaths or mortality or decedent*).tw,kf.

Concept 4:

exp Critical Care/ or exp Emergency Service, Hospital/ or exp Hospitalization/ or exp Inpatient/ or ("critical care" or "emergency room*" or ER or ED or "emergency department*" or "acute care" or "in-hospital" or "in-patient" or "in hospital" or "in patient" or inpatient or hospitali* or "intensive care" or "A and E Department" or "A and E ward" or "Casualty ward" or "casualty department" or "Emergency ward").tw,kf.
