# Supplementary material for: Understanding Responsible Development in AI-Based Clinical Prediction Models for Mortality: Protocol for a Scoping Review
Source: JMIR Res Protoc. 2026 Mar 5;15:e80325. doi: 10.2196/80325 (PMC12978964; doi:10.2196/80325)
Supplement: Multimedia Appendix 2 [file resprot-v15-e80325-s002.docx]

### Appendix II:

### A - Inclusion/Exclusion Form for **Title & Abstract Screening**

| Inclusion Criteria | |
| --- | --- |
| Is the reference in English? | y/n |
| Is the reference a journal article or conference proceedings? | y/n |
| Does the reference report research findings / results?  (not editorials, position papers) | y/n |
| Does the reference have in-hospital mortality as the primary outcome?  (Including time-to-death, risk of mortality, etc.) | y/n |
| Does the reference report AI as the source of output for the model / intervention? | y/n |
| Is the AIPM trained on human patient data or synthetic human patient data? | y/n |
| Does the reference report novel AIPMs in an acute care setting?  (In-patient unit, medical ward, ER, ICU) | y/n |
| Exclusion Criteria | |
| Is this article narrative or an editorial?  (If it’s a review and meets the above criteria, we will pass along to full text screening) | y/n |
| Does the reference report pediatric data?  (Includes neonatal, infant, pediatric) | y/n |
| Are statistical methods the only provider of mortality predictions? | y/n |

### B - Inclusion/Exclusion Criteria for **Full-Text Screening**

| Inclusion Criteria | |
| --- | --- |
| Does the reference report research findings? | y/n |
| Does the reference report AI as the source of output for the model / intervention? | y/n |
| Does the reference have in-hospital mortality as the primary outcome?  (Including time-to-death, risk of mortality) | y/n |
| Is the AIPM trained on human patient data or synthetic human patient data? | y/n |
| Are the subjects median or mean age greater or equal to 18 years?  (Average age) | y/n |
| Does the reference report novel AIPMs in an acute care setting?  (In-patient unit, medical ward, ER, ICU) | y/n |
| Exclusion Criteria | |
| Is this article narrative or an editorial? | y/n |
| Are statistical methods the only provider of mortality predictions? | y/n |
| Is the article a **review**?  (Will exclude but will check reference lists for all relevant reviews and protocols) | y/n |
