# Supplementary material for: Understanding Responsible Development in AI-Based Clinical Prediction Models for Mortality: Protocol for a Scoping Review
Source: JMIR Res Protoc. 2026 Mar 5;15:e80325. doi: 10.2196/80325 (PMC12978964; doi:10.2196/80325)
Supplement: Multimedia Appendix 3 [file resprot-v15-e80325-s003.docx]

### Appendix III: Data charting form

Data Charting Form for Research Question 1

| **Element** | **Question (Y/N)** |
| --- | --- |
| Title | Full title of the study |
| Citation (Author, Year) | Author(s), Year of publication |
| Journal / Source | Journal or publication source |
| The model’s provided output | Temporal or Probabilistic |
| Prediction Unit | X Days / X Months |
| Sample Size |  |
| Is this a development study? | Y / N |
| How many models were developed? |  |
| Is this a validation study? | Y / N |
| Source of Data | Trial, Registry, Cohort, EMR |
| Use of Synthetic Data | Y / N |
| Model Type |  |
| Machine Learning Technique | Classification, Neural Networks, Natural Language Processing |
| What socio-demographic information was captured? | Ethnicity, Race, Age, Language, Sex, Gender, Disability, Employment |
| How was the AI model trained? | Supervised or Unsupervised or Validated |
| Performance Metrics | accuracy, AUC-ROC, sensitivity, specificity, PPV, NPV, discrimination and calibration, precision recall curves |
| Comparative Study | Y / N |
| Compared performance to? | CDS, Clinical Rating Tools |
