# Supplementary figures and images for: Understanding Responsible Development in AI-Based Clinical Prediction Models for Mortality: Protocol for a Scoping Review
Source: JMIR Res Protoc. 2026 Mar 5;15:e80325. doi: 10.2196/80325 (PMC12978964; doi:10.2196/80325)

### Appendix IV: Decision tree for AI model eligibility


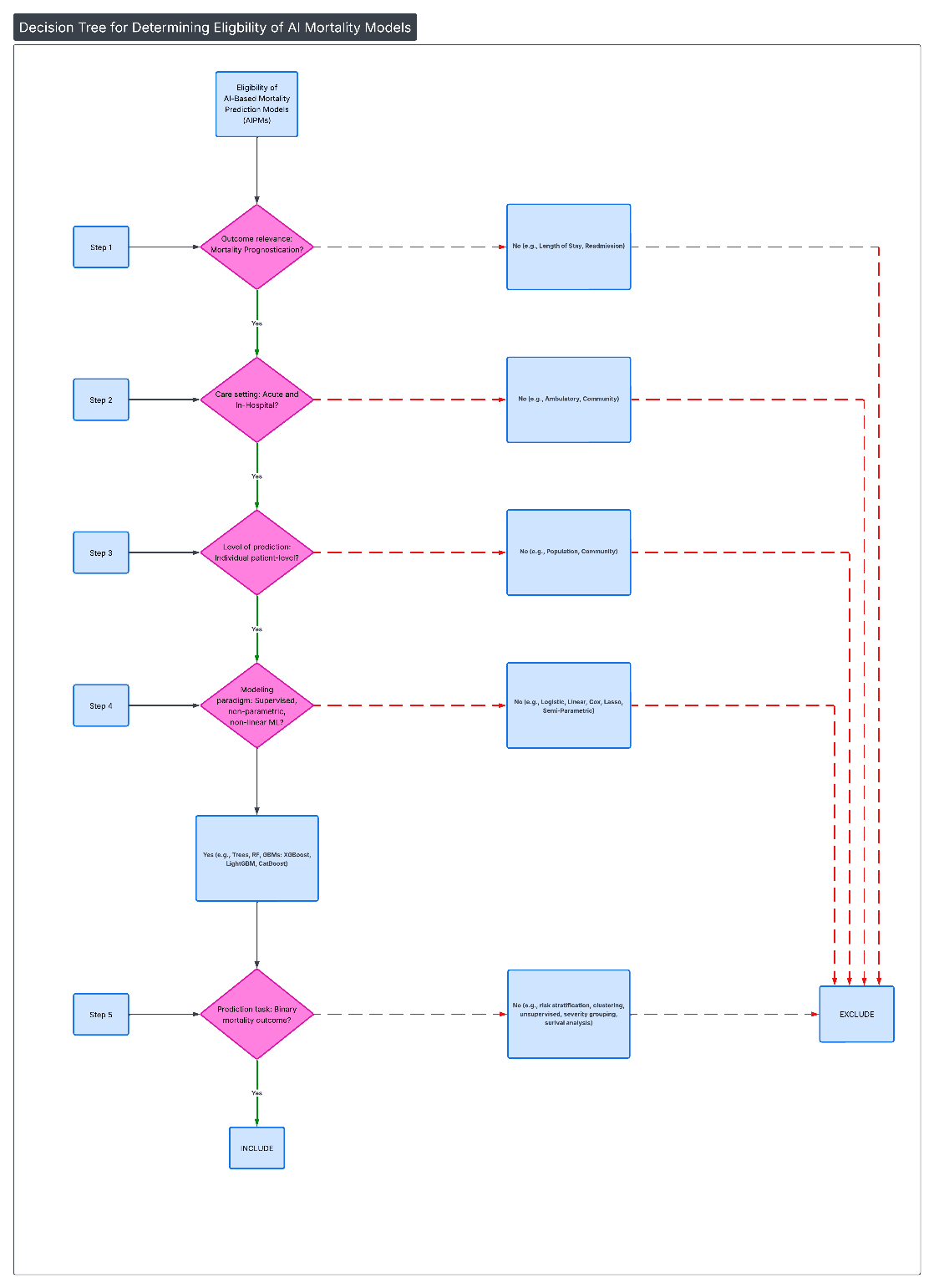

Supplement: Multimedia Appendix 4 [file resprot-v15-e80325-s004.docx]
